# Effect of insect tea on D‐galactose‐induced oxidation in mice and its mechanisms

**DOI:** 10.1002/fsn3.1278

**Published:** 2019-11-17

**Authors:** Kai Zhu, Xiaofei Zeng, Fang Tan, Wenfeng Li, Chong Li, Yaru Song, Xin Zhao

**Affiliations:** ^1^ Chongqing Collaborative Innovation Center for Functional Food Chongqing University of Education Chongqing China; ^2^ Chongqing Engineering Research Center of Functional Food Chongqing University of Education Chongqing China; ^3^ Chongqing Engineering Laboratory for Research and Development of Functional Food Chongqing University of Education Chongqing China; ^4^ Department of Cardiothoracic Surgery The First Affiliated Hospital of Chengdu Medical College Chengdu China; ^5^ Department of Public Health Our Lady of Fatima University Valenzuela Philippines; ^6^ School of Life Science and Biotechnology Yangtze Normal University Chongqing China

**Keywords:** antioxidant, D‐galactose, gene, insect tea, mice

## Abstract

Insect tea is a traditional Chinese drink that contains abundant bioactive substances. In this study, the preventive effect of Insect tea on D‐galactose‐induced oxidation in mice was studied. The serum, liver, and spleen of mice were measured by biochemical and molecular biological methods, which showed that Insect tea could increase the biochemical indexes of the thymus, brain, heart, liver, spleen, and kidney in mice with induced oxidative damage. Insect tea can increase the levels of SOD (superoxide dismutase), GSH‐Px (glutathione peroxidase), and GSH (glutathione) and decrease the levels of MDA (malondialdehyde) in the serum, liver, and spleen of mice with oxidative damage. Pathological observation also confirmed that Insect tea could inhibit oxidative damage of the liver and spleen tissue caused by D‐galactose in mice. Further molecular biological experiments also showed that Insect tea could upregulate the mRNA and protein expression of Cu/Zn‐SOD (cuprozinc‐superoxide dismutase), Mn‐SOD (manganese superoxide dismutase), CAT (catalase), HO‐1 (heme oxygenase‐1), Nrf2 (nuclear factor‐erythroid 2 related factor 2), γ‐GCS (γ‐glutamylcysteine synthetase), and NQO1 (NAD(P)H dehydrogenase [quinone] 1) in the liver and spleen of oxidized mice. Insect tea has a good preventive effect on D‐galactose‐induced oxidation in mice, and the effect is better than vitamin C, an antioxidant. Insect tea is rich in isochlorogenic acid A, quercetin, rutin, hesperidin, neochlorogenic acid, and cryptochlorogenic acid. The combination of these bioactive substances has good antioxidant effects. Thus, Insect tea is a functional food with a good antioxidant effect that has value for future development and utilization.

## Graphical abstract

Insect tea is a traditional Chinese drink that contains abundant bioactive substances. In this study, we found that Insect tea was a functional food with a good antioxidant effect that has value for future development and utilization.

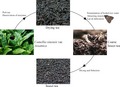

## 1 INTRODUCTION

**Figure 1:**
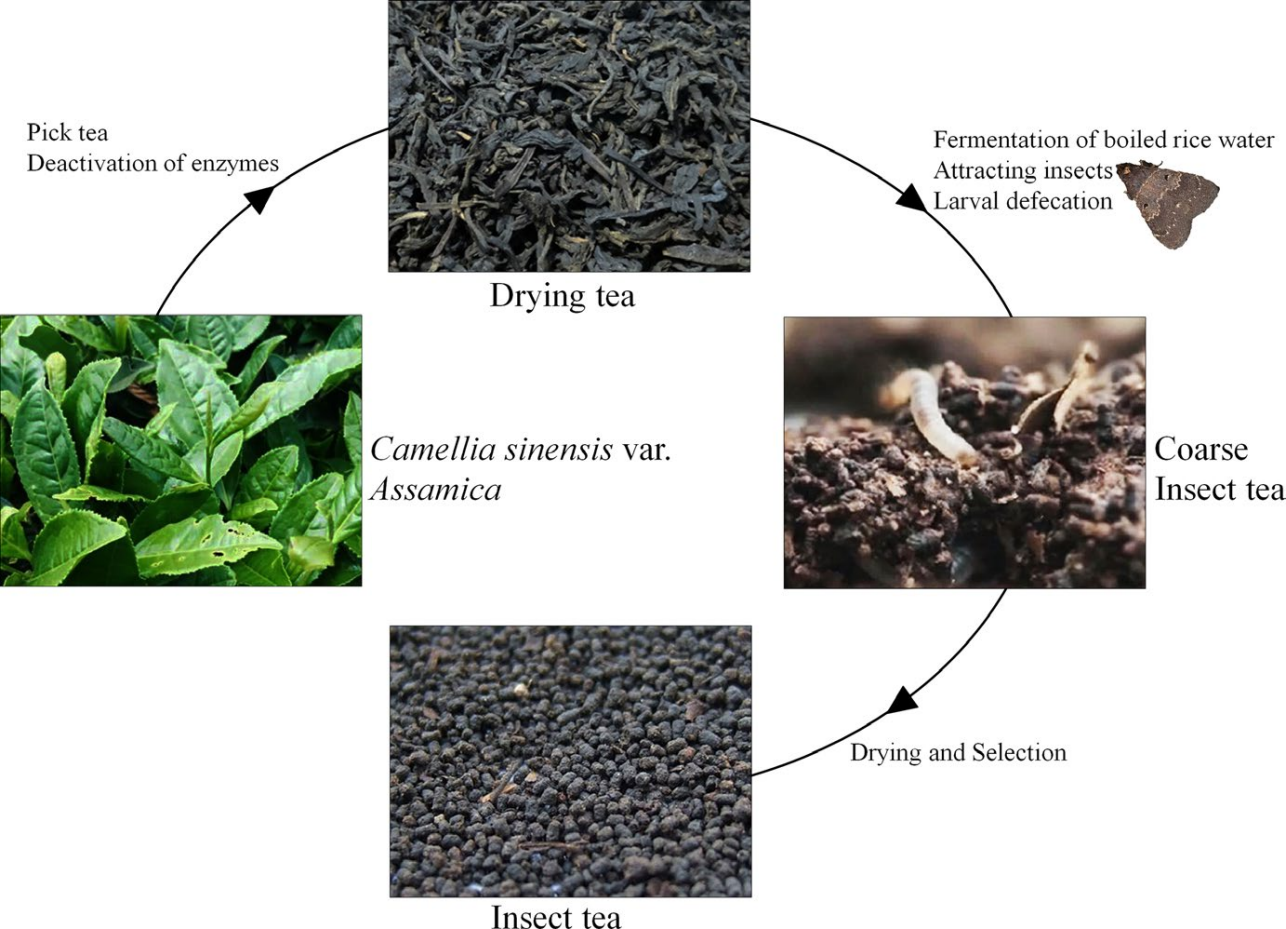
Production process of Insect tea

Insect tea is a type of special tea beverage that is produced in China; Insect tea is different from ordinary tea, as it is healthy or medicinal tea. It is a special tea beverage made by insects that is excreted in insect droppings after eating tea leaves (Li & Zhou, 2005). *Hydrillodes morose Butler*, *Nodarianiphona Butler*, *Aglossa dimidiate Haworth*, *Herculia glaucinalis L*., and *Fujimacia bicoloralis Leech* have been found to produce Insect tea (Figure 1). The larvae of these insects feed on the leaves of Kuteng tea, Chemical Tree Baije, *Trifolium pratense,* and other plants and excrete droppings. After collection, the series of processed products represent caterpillar tea products (Jiang, 2000). Insect tea contains 17 mineral elements, including K, Mg, Ca, Na, Fe, Mn, and Zn, among which approximately 10 elements are essential trace elements in the human body, and the contents of Fe, Zn, Ca, and Mg are higher than the levels of some famous teas (Yang & Li, 2011). Additionally, different Insect teas are rich in crude protein, crude fiber, fat, tea polyphenols, caffeine, sugar, vitamins, and amino acids (Guo, Xu, Wen, Huang, & Wang, 2008; Zhou, Feng, Zhu, & Zhao, 2015). Insect tea, as a traditional drink and Chinese medicine, has the functions of reducing and eliminating fever, detoxifying, strengthening the stomach, and helping digestion. It has good effects on diarrhea, epistaxis, gingival bleeding and hemorrhoid bleeding (Feng, Luo, & Zhao, 2013).

Oxygen stress occurs gradually and causes persistent damage to individuals. Oxidative stress will lead to and aggravate many diseases, including hypertension, type 2 diabetes, atherosclerosis, and dementia (Buford, 2016; Chard et al., 2017; Kitada, Ogura, & Koya, 2016). Excessive redox‐active free radicals can cause oxidative damage of biological macromolecules, leading to oxidative stress in the body, accompanied by the occurrence and development of random oxidative stress, increased production of hydrogen peroxide by mitochondria, and increased oxidative damage in the body (Rao, 2009). Redox regulation is an important issue in the study of oxidative stress. Maintaining redox balance and regulating redox‐related genes are new strategies to alleviate oxidative stress (Hohensinner et al., 2018). D‐galactose is a commonly used senescent agent in research that can be used to establish an oxidative stress animal model. A small amount of D‐galactose can be converted into glucose and will participate in metabolism, but a large amount of D‐galactose will lead to the disorder of cell metabolism, changes in the activity of oxidase in tissues and cells, and the production of many superoxide anions and oxidative products, resulting in oxidative damage to the structure and function of biological macromolecules and ultimately leading to oxidative stress (Li et al., 2016). The oxidation model of D‐galactose was established to verify the antioxidant effect of antioxidant active substances. It has been gradually applied to the research and development of antioxidant health products.

Studies have shown that natural foods have strong antioxidant and free radical scavenging abilities due to their structural characteristics. Phenolic hydroxyl structures, especially those in catechol or pyrogallol, are easily oxidized quinone structures. They have a strong ability to capture free radicals such as reactive oxygen species, including lipid free radicals produced by oxidation, which can reduce or prevent oxidation in tissues (Li, Xia, Yang, & Zhong, 2013). In animal and human clinical studies, natural foods have good antioxidant effects and can protect the body from oxidative stress‐induced damage (Carluccio et al., 2003; Li et al., 2013). Particularly, for SOD, GSH‐Px, and CAT, which are important antioxidant enzymes in the body, natural foods can effectively increase their vitality in the body and play an antioxidant role (Sharma et al., 2016).

In this study, effective active substances were extracted from Insect tea and applied to mice with D‐galactose‐induced oxidative damage. The effects of Insect tea extract on the serum and tissues of mice with D‐galactose‐induced oxidative damage were observed. The mechanism of Insect tea extract in preventing oxidation was elucidated by detecting oxidation‐related genes, which provided a theoretical basis for further human research and the industrial development of Insect tea extract.

## 2 MATERIALS AND METHODS

### 2.1 Extraction of Insect tea

Five hundred grams of Insect tea was crushed into a powder, and 50 ml of 5% ethanol solution (volume ratio) was added for extraction (90°C, 30 min). The extract was amalgamated twice and repeated and then was subjected to rotary evaporation to obtain Insect tea (Zhang et al., 2018).

### 2.2 High‐performance liquid chromatography (HPLC)

The reference substances of isochlorogenic acid A, isochlorogenic acid B, isochlorogenic acid C, quercetin, rutin, kaempferin, neochlorogenic acid, hesperidin, and cryptochlorogenic acid were precisely weighed and added to 2 ml of chromatographic methanol. The reference substances were fully shaken, and the reference substance reserve solution was obtained. The liquid phase conditions were as follows: mobile phase A: 0.5% acetic acid water; mobile phase B: acetonitrile; flow rate: 0.5 ml/min; chromatographic column: HyperSep C18 column (Five micron, 4.6 × 150 mm, Thermo Fisher Scientific, Inc.); injection volume: 10 µl; column temperature: 30°C; detection wavelength: 280 nm; and gradient conditions: 0–30 min, 12%–45% (acetonitrile); 30–35 min, 45%–100% (acetonitrile); and 35–40 min, 100% (acetonitrile). The composition of Insect tea extract was determined by using the UltiMate3000 HPLC System (Thermo Fisher Scientific, Inc.).

### 2.3 Animal oxidation experiment

Forty SPF‐grade 6‐week‐old ICR mice (Chongqing Medical University) were fed for one week, and then, the mice were divided into four groups: normal, model, Insect tea, and vitamin C. Each group consisted of 10 mice, five male and five female. During the first four weeks, the mice in the normal and model groups were only fed with diet and drinking water. The mice in the Insect tea group and vitamin C group were fed with Insect tea extract and vitamin C at a daily concentration of 100 mg/kg by gavage. Four weeks later, D‐galactose was injected intraperitoneally into each mouse of the model, Insect tea and vitamin C groups at a concentration of 120 mg/kg per day for 6 weeks. At the same time of D‐galactose injection, the mice in the Insect tea group and vitamin C group received intragastrically administered Insect tea and vitamin C at the same concentration for 6 weeks; after 24 hr of fasting, all the mice were sacrificed (Li et al., 2018). Blood was taken from the heart and liver for follow‐up experiments. The indexes of the thymus, brain, heart, liver, spleen, and kidney were measured. The organ index was equal to the organ mass (g)/body mass of mice (kg) ×100.

### 2.4 Determination of the MDA content and SOD and GSH‐Px activities in serum and liver tissues

The obtained mouse plasma was centrifuged at 730 × *g* for 10 min, and then, the upper serum layer was taken. The content of MDA and the activities of SOD and GSH‐Px in the serum were determined by using commercial kits (Nanjing Jiancheng Bioengineering Institute). A 10% homogenate was prepared from the liver of mice and was centrifuged at 730 × *g* for 10 min. The supernatant was taken to determine the MDA content and SOD and GSH‐Px activities in liver tissue using commercial reagents (Nanjing Jiancheng Bioengineering Institute).

### 2.5 Pathological observation of liver and spleen tissues

Next, 0.5‐cm^2^ liver and spleen tissues from the mice were removed and fixed in 10% formalin solution for 48 hr. The skin, liver, and spleen tissues were dehydrated, rendered transparent, waxed, embedded and sectioned, and then were stained with H&E. The morphological changes in the tissues were observed under an optical microscope (BX43, Olympus).

### 2.6 Quantitative PCR (qPCR) assay

The liver and spleen tissues of mice were crushed, and then, RNAzol was used to extract total RNA from the tissues. The total RNA concentration was diluted to 1 μg/μl, and 5 μl of this diluted total RNA solution was removed and subjected to reverse transcription to obtain cDNA. Next, 2 μl of DNA template, 10 μl of SYBR Green PCR Master Mix (Thermo Fisher Scientific, Inc.), and 1 μl each of forward and reverse primers (Table 1) were mixed and reacted at 95°C for 60 s, followed by 40 cycles of 95°C for 15 s, 55°C for 30 s, and 72°C for 35 s; finally, the DNA was detected at 95°C for 30 s and 55°C for 35 s, and GAPDH was used as an internal reference. The 2^−ΔΔCt^ method was used to calculate the relative expression of genes (SteponePlus, Thermo Fisher Scientific, Inc.) (Zhang et al., 2018).

**Table 1:** Sequences of primers used in this study

Gene Name	Sequence
Cu/Zn‐SOD	Forward: 5'‐AACCAGTTGTGTTGTCAGGAC‐3'
	Reverse: 5'‐CCACCATGTTTCTTAGAGTGAGG‐3'
Mn‐SOD	Forward: 5'‐CAGACCTGCCTTACGACTATGG‐3'
	Reverse: 5'‐CTCGGTGGCGTTGAGATTGTT‐3'
CAT	Forward: 5'‐ GGAGGCGGGAACCCAATAG‐3'
	Reverse: 5'‐ GTGTGCCATCTCGTCAGTGAA‐3'
HO‐1	Forward: 5'‐ACAGATGGCGTCACTTCG‐3'
	Reverse: 5'‐TGAGGACCCACTGGAGGA‐3'
Nrf2	Forward: 5'‐CAGTGCTCCTATGCGTGAA‐3'
	Reverse: 5'‐GCGGCTTGAATGTTTGTC‐3'
γ‐GCS	Forward: 5'‐GCACATCTACCACGCAGTCA‐3'
	Reverse: 5'‐CAGAGTCTCAAGAACATCGCC‐3'
NQO1	Forward: 5'‐CTTTAGGGTCGTCTTGGC‐3'
	Reverse: 5'‐CAATCAGGGCTCTTCTCG‐3'
GAPDH	Forward: 5'‐AGGTCGGTGTGAACGGATTTG‐3'
	Reverse: 5'‐GGGGTCGTTGATGGCAACA‐3'

Abbreviations: CAT, catalase; Cu/Zn‐SOD, cuprozinc‐superoxide dismutase; GAPDH, glyceraldehyde‐3‐phosphate dehydrogenase; HO‐1, heme oxygenase‐1; Mn‐SOD, manganese superoxide dismutase; NQO1, NAD(P)H dehydrogenase [quinone] 1; Nrf2, nuclear factor‐erythroid 2 related factor 2; γ‐GCS, γ‐glutamylcysteine synthetase.

### 2.7 Western blot analysis

Samples of liver and spleen adipose tissue (100 mg) and 1 ml of RIPA (Thermo Fisher Scientific, Inc.) were homogenized at 2,190 × *g* at 4°C for 5 min and were centrifuged at 2,190 × *g* 4°C for 15 min. The intermediate protein layer solution was extracted. The BCA protein quantitative kit (Thermo Fisher Scientific, Inc.) was used to quantify the protein. The samples of each group were diluted to 50 µg/ml, and then, the diluted protein was mixed with sample buffer at 4:1 and heated at 100°C for 5 min. Next, acrylamide, resolving buffer, stacking buffer, distilled water, 10% APS, and TEMED (Thermo Fisher Scientific, Inc.) were proportionally mixed to form an SDS‐PAGE separating and concentrating glue, which was poured into the running rubber board for use. The prestained protein ladder and samples were placed into the sample hole of the rubber sheet, and then, the SDS‐PAGE glue containing protein was subjected to vertical gel electrophoresis for 50 min. The PVDF membrane (Thermo Fisher Scientific, Inc.) was activated by methanol for 1 min, and then, the transmembrane was sealed for 1 hr by 1 × TBST solution containing 5% skim milk. After closure, the PVDF membrane was washed with 1 × TBST, and the primary antibody was incubated at 25°C for 2 hr. After washing five times with 1 × TBST, the secondary antibody was incubated at 25°C. Finally, Supersignal West Pico PLUS was used to spray the PVDF film, followed by placement in iBright FL1000 (Thermo Fisher Scientific, Inc.) for observation (Li et al., 2018).

### 2.8 Statistical analysis

The serum and tissue determination experiments of each mouse were performed three times in parallel, and then, the mean values were taken. Next, SAS 9.1 statistical software was used to analyse the data. One‐way ANOVA was used to analyse whether significant differences occurred among groups of data at the level of *p* < .05.

## 3 RESULTS AND DISCUSSION

### 3.1 Organ index

Table 2 reveals that the indexes of thymus, brain, heart, liver, spleen, and kidney of normal mice were the highest, while the index of organs of model mice is the lowest. Insect tea extract and vitamin C could significantly (*p* < .05) increase the organ index of oxidative damage‐induced mice. At 100 mg/kg, the effects of Insect tea extract were significantly (*p* < .05) better than those of vitamin C. Therefore, Insect tea can inhibit the decline in the organ index caused by tissue atrophy. The organ weight and organ index of animals are indicators to study the state of animal body. The changes in the organ weight and organ index can reflect the state of the organism subjected to oxidative stress. When the organism is under oxidative stress, the thymus and brain will atrophy more obviously than other organs (Tang & He, 2013). The liver and kidney are the main metabolic organs in mice. Decreases in their weight and organ index directly affect the metabolic ability of the organism. At the same time, the liver is one of the immune organs of animals. A change in the liver index indicates that the immunity of the organism is also affected (Khan, Singer, & Vaughan, 2017). The spleen is related to the cellular immune system in the body and plays an important role in immune mechanism. A decline in the quality of the spleen indicates organ atrophy, which can reduce its immune function. Therefore, determining the spleen organ index can directly reflect the structural changes in organs and their functions, which has important reference value to evaluating the success of the establishment of an oxidative stress mouse model (Manini, 2010). The results of this study also showed that D‐galactose could reduce the visceral index of mice induced by oxidative damage. Insect tea could alleviate the decrease in the visceral index of mice induced by D‐galactose, thus preventing the oxidative damage in mice, and the effect was better than that of vitamin C.

**Table 2:** Organ index of mice in each group

Group	Thymus index	Brain index	Cardiac index	Liver index	Spleen index	Kidney index
Normal	0.31 ± 0.03^a^	5.09 ± 0.18^a^	3.51 ± 0.15^a^	24.05 ± 0.48^a^	1.62 ± 0.08^a^	4.43 ± 0.32^a^
Model	0.14 ± 0.02^d^	3.05 ± 0.12^d^	1.74 ± 0.14^d^	17.22 ± 0.39^d^	1.02 ± 0.11^d^	2.57 ± 0.18^d^
Insect tea	0.26 ± 0.02^b^	4.26 ± 0.20^b^	2.83 ± 0.13^b^	21.31 ± 0.41^c^	1.39 ± 0.07^b^	3.72 ± 0.22^b^
Vitamin C	0.21 ± 0.02^c^	3.68 ± 0.16^c^	2.47 ± 0.14^c^	19.02 ± 0.36^c^	1.21 ± 0.08^c^	3.12 ± 0.19^c^

Note

Values represent the mean ± standard deviation. Mean values with different superscript letters (a–d) in the same row are significantly different (*p* < .05). Vitamin C: mice treated with 100 mg/kg of vitamin C; Insect tea: mice treated with 100 mg/kg of Insect tea extract.

### 3.2 SOD, GSH‐Px, GSH, and MDA levels in serum

Tables 3, 4, and 5 show that SOD and GSH‐Px in the serum, liver, and spleen of mice in the normal group were the strongest, while the MDA level was the lowest; the mice in the model group showed the opposite trend. The activities of SOD, GSH‐Px, and GSH in mice increased significantly (*p* < .05), while the level of MDA decreased significantly (*p* < .05), and the effects of Insect tea extract were significantly (*p* < .05) better than those of vitamin C. SOD is a metalloenzyme that widely exists in the biological world, and it is the key line of defense against the toxicity of reactive oxygen species in various organisms. SOD is effective in preventing and treating diseases related to superoxide free radicals. When superoxide anion radicals are produced excessively or the SOD concentration is low, excessive superoxide anion will cause oxidation (Lee, Hyun, Jenner, & Halliwell, 2001). GSH is an important antioxidant in mammals that can scavenge the free radicals produced in cells, thereby reducing the damage of the cell membrane caused by the formation of reactive oxygen species through lipid peroxidation (Berndt & Lillig, 2017). GSH is an important antioxidant in mammals that can scavenge the free radicals produced in cells, thereby reducing the damage of the cell membrane caused by the formation of reactive oxygen species through lipid peroxidation (Berndt & Lillig, 2017). GSH is directly or indirectly involved in many life activities of microbial cells. One of the most important roles of GSH is to build a strong defense line against oxygen stress together with related metabolic enzymes. GSH reduction is composed of gamma‐glutamate‐cysteine ligase (GSH1), glutathione synthetase (GSH2), glutathione reductase (GR), glutathione peroxidase (GPx), and NADPH. The interaction of various factors in the system can inhibit oxidative stress reaction of cells and slow down oxidation (Vázquez‐Medina, Zenteno‐Savín, Forman, Crocker, & Ortiz, 2011). MDA is a lipid peroxide formed by oxidation. The level of MDA in vivo also directly reflects the degree of oxidation (Hosen, Islam, Begum, Kabir, & Howlader, 2015). Based on these results, it can be seen that Insect tea can regulate oxidative regulation factors in serum.

**Table 3:** Levels of SOD, GSH‐Px, GSH, and MDA in the serum of mice

Group	SOD (U/mL)	GSH‐Px (U/mL)	GSH (mg/L)	MDA (nmol/mL)
Normal	231.28 ± 7.53^a^	211.03 ± 6.63^a^	48.02 ± 3.87^a^	3.25 ± 0.42^d^
Model	63.89 ± 4.52^d^	75.39 ± 4.82^d^	12.10 ± 1.36^d^	42.18 ± 3.55^a^
Insect tea	183.24 ± 6.71^b^	153.72 ± 5.83^b^	35.36 ± 2.58^b^	10.36 ± 0.92^c^
Vitamin C	131.08 ± 8.62^c^	118.36 ± 5.30^c^	24.71 ± 2.66^c^	19.35 ± 1.24^b^

Note

Values represent the mean ± standard deviation.

Mean values with different superscript letters (a–d) in the same row are significantly different (*p* < .05). Vitamin C: mice treated with 100 mg/kg of vitamin C; Insect tea: mice treated with 100 mg/kg of Insect tea extract.

**Table 4:** Levels of SOD, GSH‐Px, GSH, and MDA in the liver of mice

Group	SOD (U/mg prot)	GSH‐Px (U/mg prot)	GSH (mg/g prot)	MDA (nmol/mg prot)
Normal	93.88 ± 2.79^a^	206.26 ± 9.72^a^	9.33 ± 0.42^a^	1.05 ± 0.08^d^
Model	23.18 ± 1.45^d^	78.46 ± 4.66^d^	3.09 ± 0.28^d^	9.24 ± 0.21^a^
Insect tea	77.28 ± 2.38^b^	146.97 ± 6.90^b^	7.16 ± 0.31^b^	3.22 ± 0.18^c^
Vitamin C	58.69 ± 3.02^c^	117.72 ± 6.23^c^	5.33 ± 0.24^c^	5.01 ± 0.22^b^

Note

Values represent the mean ± standard deviation.

Mean values with different superscript letters (a–d) in the same row are significantly different (*p* < .05). Vitamin C: mice treated with 100 mg/kg of vitamin C; Insect tea: mice treated with 100 mg/kg of Insect tea extract.

**Table 5:** Levels of SOD, GSH‐Px, GSH, and MDA in the spleen of mice

Group	SOD (U/mg prot)	GSH‐Px (U/mg prot)	GSH (mg/g prot)	MDA (nmol/mg prot)
Normal	84.32 ± 2.41^a^	123.57 ± 8.74^a^	6.93 ± 0.25^a^	0.52 ± 0.05^d^
Model	19.62 ± 1.25^d^	41.23 ± 2.89^d^	2.06 ± 0.12^d^	4.88 ± 0.31^a^
Insect tea	68.14 ± 2.53^b^	97.36 ± 6.29^b^	5.17 ± 0.22^b^	1.79 ± 0.30^c^
Vitamin C	50.58 ± 2.37^c^	63.85 ± 6.34^c^	4.12 ± 0.19^c^	2.91 ± 0.26^b^

Note

Values represent the mean ± standard deviation.

Mean values with different superscript letters (a–d) in the same row are significantly different (*p* < .05). Vitamin C: mice treated with 100 mg/kg of vitamin C; Insect tea: mice treated with 100 mg/kg of Insect tea extract.

### 3.3 Pathological observation

**Figure 2:**
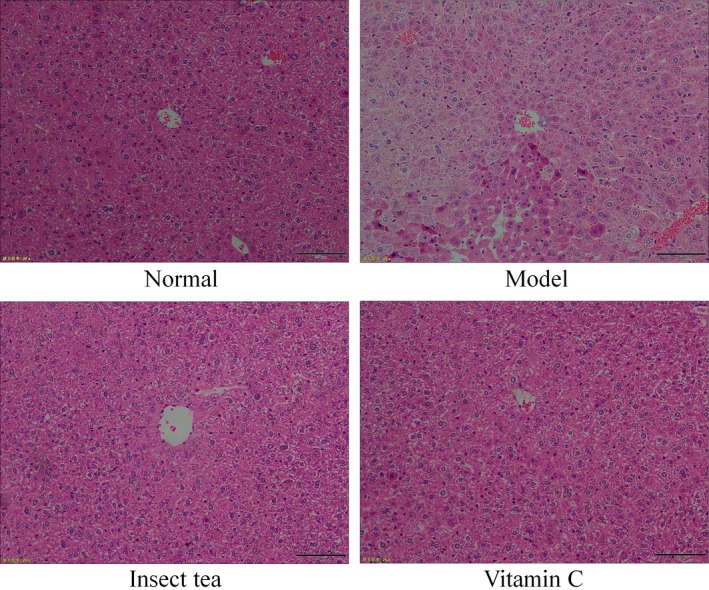
H&E pathological observation of the liver in mice. Magnification, 200×. Vitamin C: mice treated with 100 mg/kg of vitamin C; Insect tea: mice treated with 100 mg/kg of Insect tea extract

Figure 2 shows that the hepatocytes in normal mice were arranged radially around the central vein with a regular morphology and uniform size and with no aggregation of inflammatory cells. In the model group, the hepatocytes were arranged in a disorderly manner, demonstrating an irregular cell morphology, blurred cell boundaries, obvious cell swelling, incomplete nuclei in some cells, and inflammatory infiltration in many cells. Treatment with Insect tea extract and vitamin C led to a more orderly appearance of oxidized mouse liver cells and liver cords, protected the integrity of liver cells, and induced a clearer cell structure. Treatment with Insect tea extract made the morphology of oxidized mouse liver tissue cells most similar to those of normal mice.

**Figure 3:**
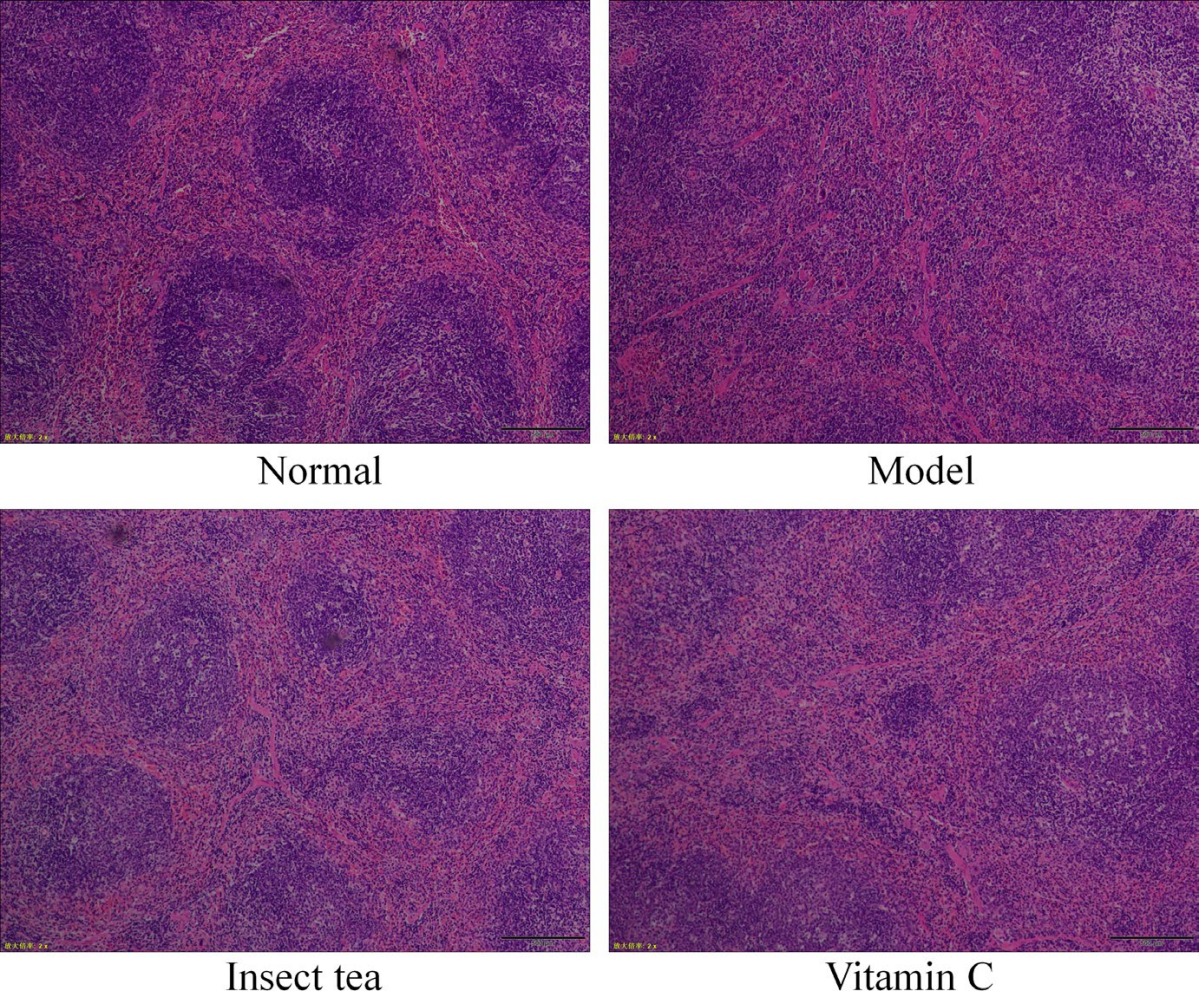
H&E pathological observation of the spleen in mice. Magnification, 20×. Vitamin C: mice treated with 100 mg/kg of vitamin C; Insect tea: mice treated with 100 mg/kg of Insect tea extract

Figure 3 shows that the spleen tissue of normal mice was clear and complete, the corticomedullary junction was clear, and the cells were arranged neatly. In the model group, the spleen tissue structure disappeared, the shape became irregular, the red medullary sinus expanded and was filled with many red cells, the number of white medullary lymphocytes decreased, the red medullary cord narrowed, and the cells were arranged sparsely. Insect tea extract effectively alleviated the changes in the spleen tissue morphology caused by oxidative damage and normalized the spleen tissue morphology of oxidative damage‐induced mice. Insect tea inhibited the pathological changes in spleen tissue caused by oxidative damage and protected the spleen; thus, Insect tea extract is more effective than vitamin C, an antioxidant.

### 3.4 Cu/Zn‐SOD, Mn‐SOD, and CAT expression in mice

**Figure 4:**
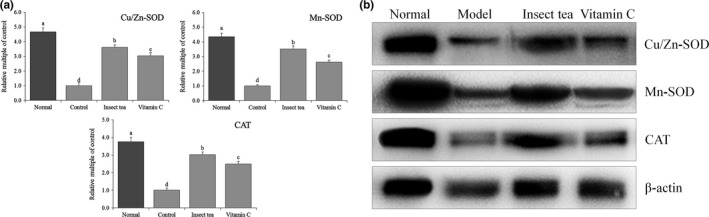
Cu/Zn‐SOD, Mn‐SOD, and CAT mRNA (a) and protein (b) expression in the liver of mice. Values represent the mean ± standard deviation. ^a–d^ Mean values with different letters in the bar are significantly different (*p* < .05). Vitamin C: mice treated with 100 mg/kg of vitamin C; Insect tea: mice treated with 100 mg/kg of Insect tea extract

**Figure 5:**
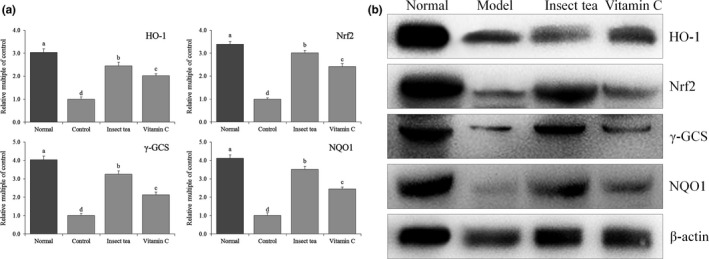
Cu/Zn‐SOD, Mn‐SOD, and CAT mRNA (a) and protein (b) expression in the spleen of mice. Values represent the mean ± standard deviation. ^a–d^ Mean values with different letters in the bar are significantly different (*p* < .05). Vitamin C: mice treated with 100 mg/kg of vitamin C; Insect tea: mice treated with 100 mg/kg of Insect tea extract

Figures 4 and 5 show that the mRNA and protein expression levels of Cu/Zn‐SOD, Mn‐SOD, and CAT in the liver and spleen of mice in the normal group were the strongest. The expression levels of Cu/Zn‐SOD, Mn‐SOD, and CAT in the liver and spleen tissues were the weakest in the model group. Compared with model mice, the expression levels of Cu/Zn‐SOD, Mn‐SOD, and CAT in the liver and spleen tissues of mice were increased significantly (*p* < .05) after treatment with Insect tea extract and vitamin C, and these expression levels in the liver and spleen tissues of these mice were closest to those of normal mice. According to the different metal auxiliary groups contained in SOD, these enzymes can be generally divided into three types: Cu/Zn‐SOD, Mn‐SOD, and Fe‐SOD. Copper/Zn‐SOD is a eukaryotic enzyme that mainly exists in the cytoplasm and chloroplast matrix of eukaryotic cells. It is also found in the blood and viscera of animals. Mn‐SOD mainly exists in the matrix of prokaryotic and eukaryotic cells with mitochondria (Bonthius, Winters, Karacay, Bousquet, & Bonthius, 2015). The activities of Cu/Zn‐SOD and Mn‐SOD in the animal body decrease when oxidative damage occurs (Kosenko et al., 2017). CAT is an antioxidant enzyme that mainly exists in erythrocytes and some tissue cells, as well as in mitochondria and the cytoplasm (Selvaratnam & Robaire, 2016). In the process of normal oxidative respiration, organisms constantly produce ROS. As a highly active molecule, it contains unpaired electrons. The enzymatic system represented by SOD can remove ROS. SOD, as the first line of defense against ROS, mainly disproportionates O_2_^−^ to H_2_O_2_. CAT can decompose H_2_O_2_, produce H_2_O_2_, and increase the oxygen content in cells (Pawlak et al., 1998). In this study, Insect tea can significantly increase the Cu/Zn‐SOD, Mn‐SOD, and CAT expression in mice, thereby effectively slowing down the oxidation caused by D‐galactose.

### 3.5 HO‐1, Nrf2, γ‐GCS, and NQO1 expression in mice

**Figure 6:**
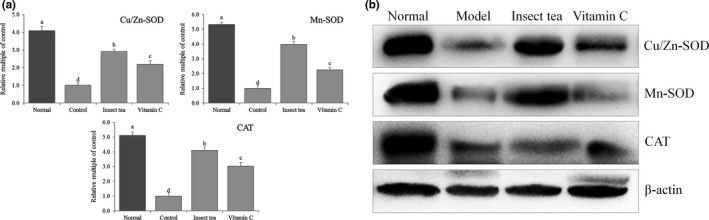
HO‐1, Nrf2, γ‐GCS, and NQO1 mRNA (a) and protein (b) expression in liver of mice. Values represent the mean ± standard deviation. ^a–d^ Mean values with different letters in the bar are significantly different (*p* < .05). Vitamin C: mice treated with 100 mg/kg of vitamin C; Insect tea: mice treated with 100 mg/kg of Insect tea extract

**Figure 7:**
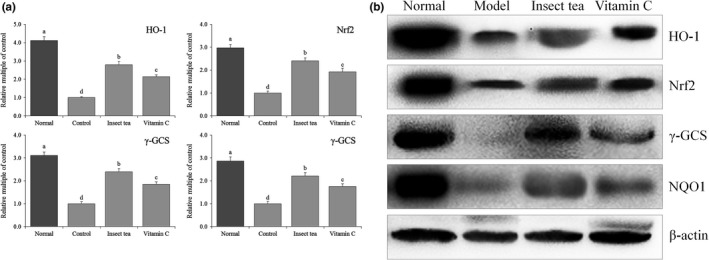
HO‐1, Nrf2, γ‐GCS, and NQO1 mRNA (a) and protein (b) expression in the spleen of mice. Values represent the mean ± standard deviation. ^a–d^ Mean values with different letters in the bar are significantly different (*p* < .05). Vitamin C: mice treated with 100 mg/kg of vitamin C; Insect tea: mice treated with 100 mg/kg of Insect tea extract

Figures 6 and 7 show that the mRNA and protein expression levels of HO‐1, Nrf2, γ‐GCS, and NQO1 in the liver and spleen of normal mice were significantly (*p* < .05) higher than those of other mice, while the expression levels of HO‐1, Nrf2, γ‐GCS, and NQO1 in the liver and spleen of model mice were the weakest. After Insect tea and vitamin C treatment, the expression levels of HO‐1, Nrf2, γ‐GCS, and NQO1 in the liver and spleen tissues of mice with D‐galactose‐induced oxidative damage were significantly (*p* < .05) increased. Moreover, the upregulation effects of Insect tea extract on these expression levels were stronger than those of vitamin C. HO‐1, as a stress protein, not only participates in heme metabolism but also extensively participates in anti‐inflammatory and antioxidant processes in vivo. It has a strong protective effect in the cardiovascular and nervous system. Studies have confirmed that HO‐1 plays an important role in cardiovascular diseases such as atherosclerosis, myocardial ischemic injury disease, hypertension, and neurological diseases, such as Alzheimer's disease, Parkinson's disease, and cerebral ischemic injury disease. The antioxidant effect of HO‐1 was more obvious (Sue et al., 2009). Nrf2 plays an important role in guaranteeing the integrity of vascular endothelium. The decline in the Nrf2 function of vascular endothelial cells in oxidative stress individuals is closely related to endothelial dysfunction. When the level of oxidative stress increases, Nrf2 is dissociated, promoting the transcription and expression of HO‐1, SOD, and CAT and thereby improving the ability of the body to understand oxygen free radicals in vivo (Hong et al., 2017). Nrf2 also can regulate gamma‐GCS. When much ROS is produced under the imbalance of oxidation/antioxidation, Nrf2 is activated, which promotes high levels of gamma‐GCS expression; thus, gamma‐GCS stimulates the synthesis and activation of GSH and plays a role as an antioxidant (Iwayama et al., 2017). When cells are under oxidative stress, Nrf2 can uncouple with Keapl, which can be activated and transferred into the nucleus, bind to ARE, regulate the expression of the downstream antioxidant enzyme gene NQO1, and enhance the tolerance of cells to oxidative stress. Therefore, regulating the Nrf2/NQO1 signaling pathway is an effective means to exert the antioxidant activity of active substances (Jiang et al., 2017). In this study, Insect tea can upregulate the expression of oxidized Nrf2, further enhancing the expression intensity of HO‐1, gamma‐GCS, and NQO1, and playing a protective role in mice with D‐galactose‐induced oxidative damage and preventing oxidative stress. The effect is better than that of vitamin C, the commonly used oxidant.

### 3.6 Constituents of insect tea

**Figure 8:**
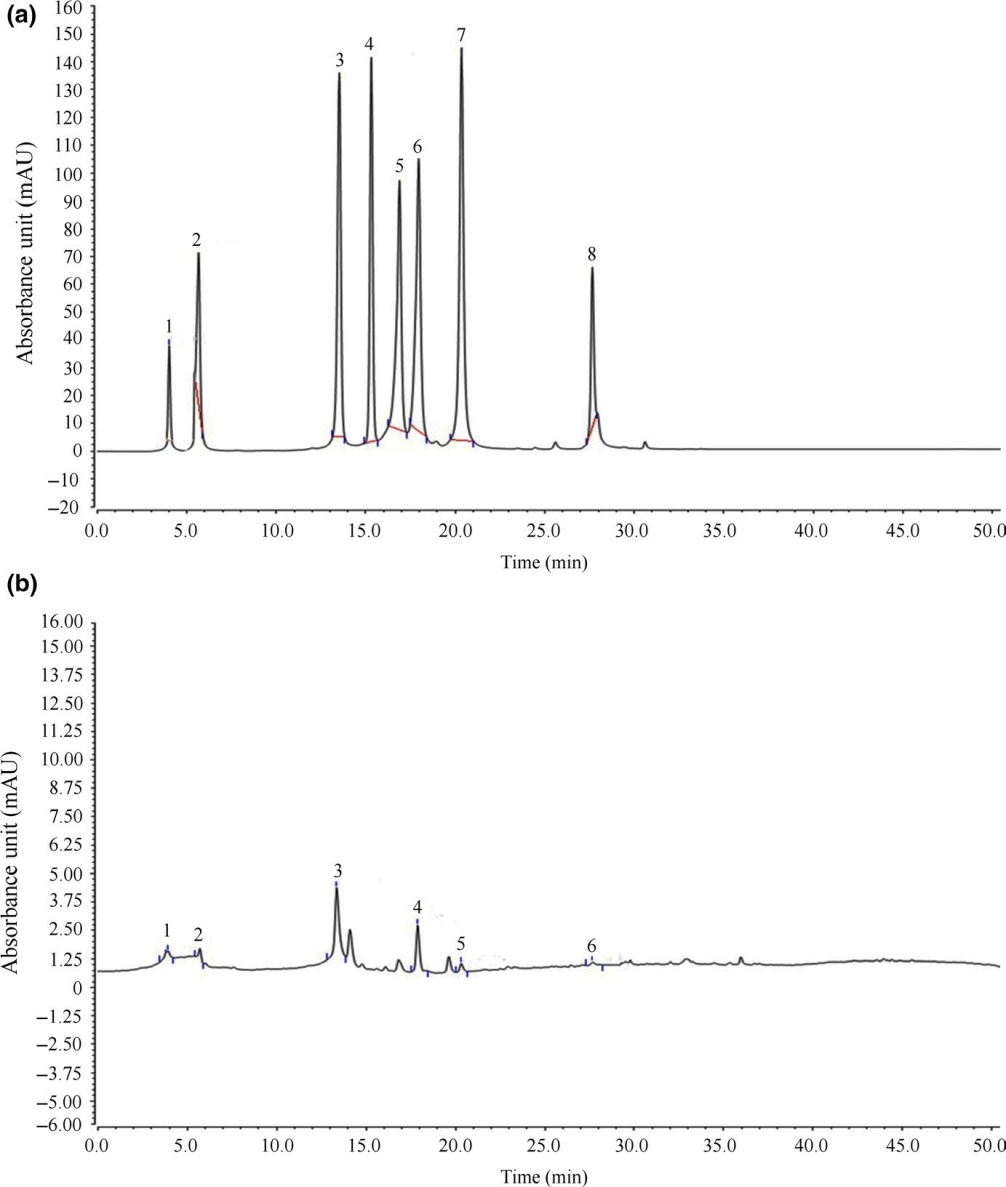
Determination of Insect tea constituents by HPLC. (a) Standard chromatograms—1: neochlorogenic acid, 2: cryptochlorogenic acid, 3: rutin, 4: kaempferin, 5: isochlorogenic acid B, 6: isochlorogenic acid A, 7: hesperidin, and 8: quercetin. (b) Insect tea chromatograms—1: neochlorogenic acid, 2: cryptochlorogenic acid, 3: rutin, 4: isochlorogenic acid A, 5: hesperidin, and 6: quercetin

Insect tea contained neochlorogenic acid, cryptochlorogenic acid, rutin, isochlorogenic acid A, hesperidin, and quercetin (Figure 8) at 0.90, 2.04, 6.43, 1.64, 1.63, and 1.59 mg/g, respectively. Phenols are natural antioxidants because they have more active phenolic hydroxyl group, which can provide hydrogen and free radicals to react to form inert products or more stable free radicals, thus interrupting or slowing down the chain reaction of free radicals (Wang et al., 2011). The results showed that the antioxidant activity of polyphenols and flavones in some natural foods is much better than that of vitamin C. The difference in the antioxidant activity of polyphenols and flavones in natural foods is not only due to the molecular structure but also to the difference in stereoconformation (Shen, Jin, Yang, & Zhao, 2000; Yilixiati & Li, 2012). Neochlorogenic acid, cryptochlorogenic acid, and isochlorogenic acid A are isomers of chlorogenic acid. They are effective phenolic antioxidants. Their antioxidant capacity is stronger than that of caffeic acid, p‐hydroxybenzoic acid, ferulic acid, syringic acid, butyl hydroxy anisole (BHA), and tocopherol. These active chlorogenic acid isomers contain R‐OH radicals, which can form hydrogen free radicals with antioxidant activity to eliminate the activity of hydroxyl radicals and superoxide anions, thus protecting tissues from oxidative damage (Wang, Xi, Fan, Cao, & Jiang, 2019). For example, isomers of chlorogenic acid (Neochlorogenic acid, cryptochlorogenic acid) have stronger activity of.OH free radical scavenging than tea polyphenols (Zhang & Wang, 2007). Rutin is a flavonoid compound and a strong oxidant for scavenging free radicals. It can terminate the chain reaction of free radicals, inhibit the peroxidation of polyunsaturated fatty acids on biofilms, remove lipid peroxidation products, and protect the integrity of biofilms and subcellular structures. Rutin plays an important role in the body (Zhang, Liu, Song, & Chen, 2003). Hesperidin is easily oxidized and destroyed, is often used as a natural antioxidant in the food industry, has antimicrobial activity, and prevents the skin from oxidative damage caused by ultraviolet radiation (Abolaji, Babalola, Adegoke, & Farombi, 2017). Quercetin is a 5‐hydroxyflavone with double bonds between the two and three sites in its molecule and two hydroxyl groups at the 37 and 47 sites. Quercetin can be used as a free group acceptor in metal chelation or oxidation of oils and fats. Additionally, quercetin can be used as an antioxidant of oils and ascorbic acid. It has also been gradually applied in functional foods with antioxidant activity (Zheng et al., 2017). Insect tea contains active substances with a strong antioxidant effect. This study also confirmed that the antioxidant effect of Insect tea at the same concentration is much stronger than that of vitamin C. Insect tea can play a preventive role in animal oxidation by containing active ingredients.

## 4 CONCLUSIONS

This study showed that Insect tea could prevent D‐galactose‐induced oxidation in mice, making the indexes of the serum, liver, and spleen in mice with D‐galactose‐induced oxidative damage similar to those observed in normal state mice, and increased levels of vitamin C with the same concentration of strong antioxidants were found. The research further confirmed that Insect tea contains abundant bioactive substances and needs further applications and study. Additionally, Insect tea can be used as a high‐quality food and medicine homologous resource rich in antioxidants. The types of effective substances in Insect tea have also been verified, and the mechanism of action has been preliminarily clarified in this study. However, further human studies are needed to confirm its mechanism more accurately, and the relationship and binding effect of various active substances need to be further studied.

## CONFLICT OF INTEREST

The authors of this manuscript state that they do not have conflict of interest to declare.

## ETHICAL APPROVAL

The protocol for these experiments was approved by the Ethics Committee of Chongqing Collaborative Innovation Center for Functional Food (201901001B, Chongqing, China).
