# Erratum

**DOI:** 10.1002/fsn3.2356

**Published:** 2021-06-11

In the article by Zhu et al. (Zhu et al., 2019), the captions for Figures 5 and 6 were reversed in the published version. The correct version of the Figure captions is provided as below.

**Figure 5.** HO‐1, Nrf2, γ‐GCS, and NQO1 mRNA (a) and protein (b) expression in liver of mice. Values represent the mean ± standard deviation. a–d Mean values with different letters in the bar are significantly different (*p* < .05). Vitamin C: mice treated with 100 mg/kg of vitamin C; Insect tea: mice treated with 100 mg/kg of Insect tea extract.

**Figure 6.** Cu/Zn‐SOD, Mn‐SOD, and CAT mRNA (a) and protein (b) expression in the spleen of mice. Values represent the mean ± standard deviation. a–d Mean values with different letters in the bar are significantly different (*p* < .05). Vitamin C: mice treated with 100 mg/kg of vitamin C; Insect tea: mice treated with 100 mg/kg of Insect tea extract.
